# Randomized Controlled Trial Testing an HIV/STI Prevention Intervention Among People Leaving Incarceration Who Were Assigned Male at Birth, Have Sex with Men and A Substance Use Disorder

**DOI:** 10.1007/s10461-025-04818-4

**Published:** 2025-07-10

**Authors:** Katrina M. Schrode, Gabriel G. Edwards, Brandon Moghanian, Robert E. Weiss, Cathy J. Reback, Charles McWells, Charles L. Hilliard, Nina T. Harawa

**Affiliations:** 1 Department of Psychiatry, Charles R. Drew University of Medicine and Science, Los Angeles, CA USA; 2 Department of Medicine, David Geffen School of Medicine, University of California, Los Angeles, Los Angeles, CA USA; 3 Center for HIV Identification, Prevention, and Treatment Services, University of California, Los Angeles, Los Angeles, CA USA; 4 Department of Biostatistics, Fielding School of Public Health, University of California, Los Angeles, Los Angeles, CA USA; 5 Friends Research Institute, Los Angeles, CA USA; 6 Center for Behavioral and Addiction Medicine, Department of Family Medicine, University of California, Los Angeles, Los Angeles, CA USA; 7 Los Angeles Centers for Alcohol and Drug Abuse, Los Angeles, CA USA; 8 Department of Research, Charles R. Drew University of Medicine and Science, Los Angeles, CA USA; 9 Department of Psychiatry and the Behavioral Sciences, Keck School of Medicine, University of Southern California, Los Angeles, CA USA

**Keywords:** HIV, Incarceration, Substance use disorder, MSM, Transgender women, PrEP

## Abstract

HIV disproportionately impacts minoritized individuals, particularly those of intersectional minoritized identities. Incarceration disproportionately impacts minoritized individuals as well, and increases HIV risk, in part due to its disruption to people’s lives, social networks, and access to care. We developed MEPS, a 6-month intervention designed to holistically support HIV prevention in men who have sex with men and transgender women leaving incarceration. We tested MEPS in a 1:1 randomized controlled trial with 208 individuals. All participants received a needs assessment and personalized wellness plan, followed by either standard of care or the MEPS intervention. MEPS integrated support from a Peer Mentor, incentives for engagement in social and health services, and a mobile app. Participants completed baseline assessments and follow-up assessments at 3, 6, and 9 months. We tested for changes in PrEP use using a group-based trajectory model, for changes in HIV and STI testing, frequent substance use and recidivism using logistic mixed models, and for changes in HCV testing and in having a regular place for care using Poisson models. MEPS participants were significantly more likely than control participants to be among those who used PrEP [AOR (95% CI) = 3.8 (1.8, 8.0)]. Recent HIV testing in the MEPS arm remained above 50% over time while decreasing in the control arm, with a significant difference between arms at 6 months [AOR (95% CI) = 3.5 (1.3, 9.5)]. There were no significant differences between arms in the other outcomes. The MEPS intervention was effective in increasing PrEP uptake and HIV testing in people leaving incarceration. Interventions that implement peer mentor support and incentives to encourage service engagement can improve engagement in HIV prevention services among populations that experience unique barriers to care. This study was registered with ClinicalTrials.gov on July 25, 2019 (NCT04036396).

**Supplementary Information:**

The online version contains supplementary material available at 10.1007/s10461-025-04818-4.

## Resumen

El VIH afecta de manera desproporcionada a personas minorizadas, especialmente a aquellas con identidades interseccionales minorizadas. La encarcelación también impacta de manera desproporcionada a estas poblaciones y aumenta el riesgo de VIH, en parte debido a la disrupción que causa en sus vidas, redes sociales y acceso a la atención médica. Desarrollamos MEPS, una intervención de seis meses diseñada para apoyar de manera integral la prevención del VIH en hombres que tienen sexo con hombres y mujeres transgénero que están saliendo de la cárcel. Probamos MEPS mediante un ensayo aleatorizado y controlado 1:1 con 208 participantes. Todos los participantes recibieron una evaluación de necesidades y un plan de bienestar personalizado, seguidos por el cuidado estándar o la intervención MEPS. MEPS integró el apoyo de un Peer Mentor (mentor entre pares), incentivos por participación en servicios sociales y de salud, y una aplicación móvil. Los participantes completaron evaluaciones al inicio y seguimientos a los 3, 6 y 9 meses. Evaluamos los cambios en el uso de PrEP mediante un modelo de trayectoria grupal; y los cambios en pruebas de VIH e ITS, uso frecuente de sustancias y reincidencia mediante modelos logísticos mixtos; así como los cambios en pruebas de hepatitis C y en tener un lugar regular de atención mediante modelos de Poisson. Los participantes del grupo MEPS tuvieron una probabilidad significativamente mayor que los del grupo control de estar entre quienes usaron PrEP [OR ajustado (IC 95%) = 3.8 (1.8, 8.0)]. Las pruebas recientes de VIH en el grupo MEPS se mantuvieron por encima del 50% con el tiempo, mientras que disminuyeron en el grupo control, con una diferencia significativa entre grupos a los 6 meses [OR ajustado (IC 95%) = 3.5 (1.3, 9.5)]. No se observaron diferencias significativas entre grupos en los otros resultados. La intervención MEPS fue eficaz para aumentar el uso de PrEP y las pruebas de VIH en personas que salen de la cárcel. Las intervenciones que implementan el apoyo de mentores pares e incentivos para fomentar la participación en servicios pueden mejorar la vinculación con servicios de prevención del VIH en poblaciones que enfrentan barreras únicas para acceder a la atención. Este estudio fue registrado en ClinicalTrials.gov el 25 de julio de 2019 (NCT04036396).

## Introduction

In the US, the greatest burden of HIV continues to fall on minoritized individuals. In 2022, 71% of new infections were among gay, bisexual, and other cisgender men who have sex with men (MSM) [1], and, based on data from 2019 to 2022, the prevalence of HIV among transgender (hereafter: trans) women was estimated at 42% [2]. The subset of those carrying multiple intersectional marginalized identities experience disproportionately elevated risk. Importantly, the disparities in HIV diagnoses between groups are not explained by differences in risk behaviors, but rather by social determinants such as income, health insurance, and racism [3, 4].

Mass incarceration has been cited as contributing to the HIV epidemic [5] and disproportionately affects sexual and gender minorities at approximately three times the rate of the general population [6]. In a study of the Veterans Aging Cohort, 40% of MSM living with HIV reported a history of incarceration, with 9% having been incarcerated in the previous year [7]. Similarly, in a longitudinal study of Black and Latino MSM in Los Angeles, among those living with HIV, 41% reported a history of incarceration and 20% reported incident incarceration during follow-up [8]. Substance use is one factor interlinked with both incarceration and risk for HIV. Recent reports indicate that 40% of people arrested, and at least 30% of people under community supervision after leaving incarceration, have a substance use disorder. Entering and leaving jail is a destabilizing experience responsible for interruptions in physical and mental health treatment, disruptions in employment and housing, and fragmenting of social networks and partnerships [5, 9, 10]. In particular, these disruptions increase the risk for HIV transmission post-release by increasing the risk for substance use and relapse, as well as the risk of returning to jail. Risk is also elevated due to interruptions in anti-retroviral treatment (ART) among those living with HIV [11]. While a limited number of interventions have been shown to improve continuity of care during reentry for individuals living with HIV [12–14], few reentry interventions have been developed for those who are at risk for HIV.

Pre-exposure prophylaxis (PrEP) is a biomedical tool that has been shown in several large randomized controlled trials to be highly effective in preventing transmission of HIV [15]. Slow increases in PrEP uptake have been the norm since its approval in 2012. In 2022, the CDC estimated that only about 36% of those who could benefit from taking PrEP were taking it, and in early 2023, this number had dropped to 25% [16]. Furthermore, estimates of uptake are much lower among non-white racial/ethnic groups compared to white individuals, even after correcting analyses for factors like age and insurance status [17, 18]. Uptake among cis and trans women have also lagged compared to MSM [18–20]. A few studies among individuals with criminal justice involvement have generally reported that while knowledge of PrEP is low, interest and stated willingness to take it is high [21–24]. However, even if an individual can access PrEP through one of the limited jail PrEP programs, multiple barriers to continuing PrEP emerge post-release [21, 24, 25].

A strategy that is increasingly common in impacting health-related behaviors is the use of peer-based interventions [26]. In peer-based interventions, participants benefit from guidance from peers with lived experience similar to their own. Peers receive specialized training about topics such as client-specific health issues, service navigation, and motivational interviewing; they also serve as role models and provide support. Peer interventions can be particularly effective for individuals from marginalized groups, who are often isolated, lacking in resources and knowledge, and subject to stigma and discrimination, all of which are associated with poorer health outcomes [27, 28]. By providing empathy and an example of success, peers can help empower participants and improve self-esteem, well-being, and self-efficacy [28–30]. The use of peers in interventions has been shown to be an effective factor in increasing uptake of health-related behaviors and services [26]. One meta-analysis showed that peer education was effective in increasing HIV testing and condom use and reducing sharing of injection drug equipment for up to 48 months after the intervention [31]. Peers may also serve a particularly important role in building trust in healthcare and service providers among participants who have experienced stigma and discrimination from these systems [32]. Several studies have shown positive attitudes toward hypothetical peer interventions focusing on PrEP [33, 34], but as of yet, there have been few tests of such interventions in vivo [35–37].

Contingency management is a behavioral economics intervention that uses positive reinforcement, often in the form of financial vouchers, to reinforce positive behaviors. Contingency management is one of the most effective behavioral interventions, most widely used for substance use treatment disorder [38–40]. Financial incentives have been an essential component of effective interventions for promoting adherence to tuberculosis prevention medication [41], HIV treatment [37], PEP use [42], and physical activity plans [43]. Despite the efficacy of contingency management interventions, challenges such as objective verification and incentive delivery can limit their adoption, but may be overcome through the use of mobile and digital technologies [44].

In recent years, the number of technology-based interventions has increased dramatically. For example, text-messaging interventions have been effective in increasing PrEP uptake [45, 46], as well as engagement in other prevention behaviors including reduced unprotected sex [47] and increased HIV testing [48]. A 2020 review of app-based interventions focused on HIV prevention found that most provided education or support for behavioral change, but only 11% facilitated linkage to care, and none supported care, including PrEP use [49].

In this randomized controlled trial (RCT), we tested the effectiveness of the novel Mobile-Enhanced Prevention Support for People Leaving Jail (MEPS) intervention in increasing HIV prevention following jail release among individuals with substance use disorder who were assigned male at birth and have sex with men. The MEPS intervention combines support from peer mentors, incentives for obtaining health-related services, and a mobile app, with the aim of improving outcomes during re-entry. As a holistic intervention, in addition to traditional prevention strategies such as PrEP and HIV testing, our outcomes included substance use and recidivism given their role as risk factors for HIV. We hypothesized that those assigned to the MEPS intervention would show increased PrEP uptake, HIV/STI/HCV testing, and access to healthcare, and reduced substance use and recidivism compared to those in the control arm.

## Methods

### Participants

Individuals assigned male at birth (including cisgender men, non-binary and trans women gender identities) who have sex with men were recruited between November 14, 2019 and December 13, 2022 from LA County. For those recruited in Men’s Central Jail (MCJ), to be eligible for the study, individuals had to (1) be housed in the protective custody K6G unit for sexual and gender minorities, (2) expect to remain in jail for at least 4 more days but no more than 3 more months, (3) screen positive for a substance use disorder, (4) be aged 18–49 years, (5) report anal or vaginal intercourse with a man or a trans woman in the 6 months prior to jail entry, (6) report no prior diagnosis of HIV, and (7) plan to reside in LA County for at least 12 months subsequent to release. Those recruited in the community had to (1) report a recent release from a jail, prison or other detention facility, (2) have sought or received substance use services since the 12 months before their most recent incarceration and fulfill criteria 4–7 listed above. Initially for those recruited in the community, release had to be within 6 months of recruitment, but this requirement was changed to 12 months in June of 2021 to increase enrollment, given that barriers to re-entry and care persisted beyond 6 months, particularly for those who were in treatment facilities for extended periods. In March 2022, we added the criterion assigned male at birth, because we were no longer recruiting from the men’s jail. One cisgender female enrolled prior to this update was excluded from analysis. Exclusion criteria were (1) not having a smartphone and being unwilling to obtain one, (2) inability to speak and understand English, and (3) lacking the reading skills necessary to use a mobile app.

All participants provided written or verbal informed consent. This study was approved by the University of California, Los Angeles Institutional Review Board (IRB# 19–000165) and Los Angeles County Department of Health Services.

### Recruitment and Screening

Participants were recruited through a variety of methods, including direct recruitment at supportive housing facilities in LA County and in MCJ, referrals from community partners, participant referrals, and flyers posted by community organizations. Due to precautions taken in response to the COVID-19 pandemic, enrollments after March 2020 occurred over the phone, except for those completed in MCJ. For direct recruitment from MCJ, individuals were randomly selected to meet with a research team member from a daily census list provided by a sheriff’s deputy or by a substance use treatment program operated by the Los Angeles Centers for Alcohol and Drug Abuse (L.A. CADA), the study’s primary community partner. Interested individuals were screened, consented and enrolled during the visit. At other facilities, individuals could call the main study line or attend an in-person event for screening, and would complete informed consent and enrollment at a later time. Enrollment and follow-up was tracked using REDCap electronic data capture tools [50].

### Participant Referrals

Currently enrolled participants were able to refer others for potential enrollment in the study. To reduce the possibility of coercion, interested referred individuals had to contact the study directly and provide a unique referral code they had received from the enrolled participant. Participants were compensated $5 for referrals, up to a total of six, and received an additional $20 for each successful enrollment, up to three. These restrictions were put in place to limit biases to the sample from over enrollment through a single network. An additional restriction was that second-degree enrollees (an individual that was referred by a referred individual) were not eligible for compensated referrals.

### Randomization and Enrollment

Participants were randomly assigned 1:1 to the MEPS (arm = 1) or control (arm = 0) condition. Assignments for the entire sample were generated in advance by the data manager, using a procedure for random allocation in SAS v9.4. The research assistants were blinded to allocations prior to enrollment. Participants selected 5 items from a list of predominantly hygiene-related items to be mailed to them as part of a Welcome Home Kit. The final step of enrollment was completion of the baseline survey.

Following enrollment, participants were mailed the Welcome Home Kit items, along with a rechargeable battery pack for their smartphone, condoms, lube, and a reloadable bank card for compensation. After the onset of the COVID-19 pandemic, hand sanitizer and masks were included in the Welcome Home Kit.

### Surveys

**Fig. 1:**
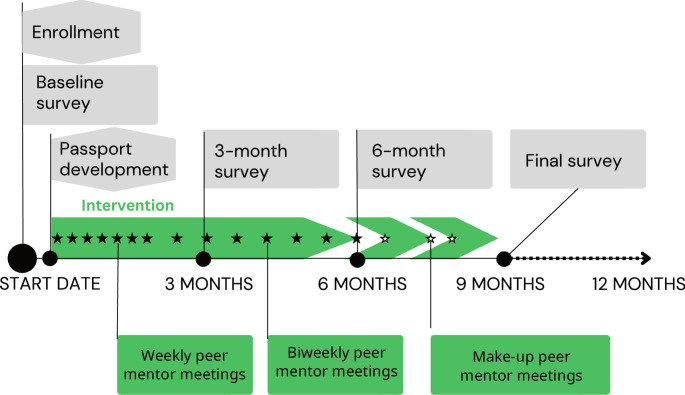
Timeline of the study. Dark circles indicate major events including enrollment and passport development and surveys, as described by associated flags. The green arrow shows the timing of the intervention relative to other events. Filled stars indicated ideal position of peer sessions, while open stars represent peer sessions delayed into the 6–9 month timeframe. The final follow-up is scheduled at 9 months, or 3 months after the last peer session

Participants completed a survey at baseline and follow-up surveys at 3 and 6 months after developing their “passport” or personalized wellness plan. An overview of the study timeline is illustrated in Fig. 1. While most passports were completed within 48 h of enrollment, there were instances where further engagement in the study was delayed. If 3 months passed without successful passport completion, the start date was set at date of enrollment, but otherwise completion of the passport signaled the start of engagement. A final follow-up survey was completed at 9 months, or 3 months following intervention completion. Surveys assessed sociodemographic factors including age, self-identified gender, race/ethnicity, and sexual identity, educational attainment, housing instability, time spent in foster care, student status, employment for wages, financial situation, history of housing instability, history of incarceration, access to healthcare and engagement in care, service needs and health behaviors. Surveys took an average of 63 (SD = 31) minutes to complete; participants were compensated $40 for baseline surveys and $50 for follow-up surveys. Surveys completed in custody were compensated $25, due to decreased participant burden. Participants received an additional $20 for completing the passport development session.

### Outcome Measures

PrEP use since last interview was measured through a series of questions. All assessments used a three-month recall period. Participants who reported ever using PrEP were asked if they were currently using PrEP and if not, when they had last used PrEP. These dates were used to determine if the participant had used PrEP in the past three months. At follow-up, participants were asked if they had used PrEP since their last assessment.

For HIV, STI, and HCV testing, we asked participants if they had ever been tested for each, and if yes, the date of their last test. We used these dates to construct binary variables indicating if the participant had been tested for HIV or STIs in the prior 3 months. For HCV testing, participants were not expected to be tested repeatedly, so a single variable was constructed whereby the participant was defined as achieving the outcome if they received testing at any point during follow-up.

To determine access to healthcare, we asked participants “In the community, when you want care for a health problem, where do you usually go?” with response options of “Doctor’s office, Kaiser, or other HMO”, “Clinic or community health center”, “Emergency room”, “Urgent care”, “Nowhere”, or “No one place.” As participants were only expected to establish a regular place for care once, similar to HCV testing, we constructed a single binary variable, regular place for care, where the participant was considered to have achieved the outcome if they responded, “Doctor’s office, Kaiser, or other HMO”, or “Clinic or community health center” at any follow-up.

Participants were asked about their frequency of using a variety of substances in the past 3 months including illicit drugs, alcohol, and marijuana. Response options were “never,” “only a few times,” “1–3 times per month,” “1–5 times per week,” and “daily”. We constructed a binary variable, frequent substance use representing use of illegal or self-identified problematic substances. Individuals were categorized as having frequent substance use if they reported at least weekly use of illegal substances. In a separate question, participants were asked what substance had caused them the most problems in their lives. If their response was marijuana or alcohol, and they reported at least weekly current use of that substance, then they were also categorized as having the outcome.

To assess recidivism, at each follow-up assessment participants were asked if they had returned to jail or prison since their last assessment, and we constructed a binary variable recidivism, with value = 1 if they had returned to jail or prison since their last interview, and 0 otherwise.

### Passport Development

Passport development was completed following enrollment (Fig. 1). These participant-centered wellness plans were based on a needs assessment generated from responses to the baseline survey and a discussion with participants about their priorities and concerns. All passports included concrete goals and recommended steps for achieving them, along with customized referrals for assistance. Initially, passports were developed with participants by a member of the L.A. CADA staff at the facility where the participant was located (MCJ or L.A. CADA residential housing). Beginning July of 2020, the Clinical Supervisor of the study’s Peer Mentors began completing passport development with all participants over the phone. During the call, the passport developer guided the participant in identifying goals in the immediate, near, and long-term time frames. Those randomized to the MEPS intervention also selected one of the available peer mentors based on short biographies. The passport development process took approximately 45 min to an hour.

### Study Arms

The study design has been described previously [51]. Briefly, all participants in the study received Welcome Home Kits, completed passport development and baseline and follow-up assessments as described in the preceding sections. Additionally, all participants were asked to check in with study staff at least once per month for an incentive beginning the month after enrollment.

### Control Arm

The control arm was a standard of care control. Initial recruitment was among those enrolled in a jail-based substance use treatment and re-entry transition program. After the majority of enrollment became community-based, most participants were in substance use treatment programs at one of our partner facilities when enrolled.

### MEPS Arm

Those assigned to the MEPS arm received the MEPS intervention in addition to the standard of care they were otherwise receiving. The MEPS intervention combined the use of peer mentors, monetary incentives, and a mobile app, Geopass. Peer mentors were recruited from the community, identified as either a gender and/or sexual minority individual, and had lived experience regarding a history of substance use and/or incarceration. Mentors were selected based on having similar backgrounds and experiences to participants and having proactively engaged with service systems in their own lives, including those for health care, recovery, and employment. Over the course of the study, 8 peer mentors were employed: 5 cisgender men, 2 trans women, and 1 trans man; 6 identified as African American, 1 as Latina, and 1 as White. Most of the participants worked with 2 African American cisgender men and 1 trans Latina, due to the timing of their employment with the study. Training for peer mentors lasted approximately 48 h and involved training on research with human subjects, basic counseling skills, motivational interviewing, HIV, Hepatitis C, STIs, SUDs, PrEP/PEP, and teach-back and role play scenarios to practice and demonstrate skills acquired.

The intervention consisted of 14 peer sessions. For the first 7 weeks of MEPS, participants met with their peer mentors weekly, at which point they transitioned to a bi-weekly schedule until the end of the intervention (Fig. 1). Prior to study start, the investigators developed the schedule of intervention sessions, including themes to be discussed during each session. Themes included items such as “Build rapport,” “Reinforce willingness to change risk behaviors,” “Explore the need for and strategies involved in the development of a positive social support network,” and “Explore challenges to or successful avoidance of threats to SUD treatment goals.” To help guide the sessions, peer mentors were provided with checklists describing each session’s planned themes and expected time allotments. Peer mentors also discussed progress toward passport goals and were afforded the flexibility to tailor additional discussion topics to the needs of each participant. Peer sessions could be completed in 6 months or continue up to 9 months, depending on participant availability as short jail stays or in-patient treatment sometimes restricted their engagement. One additional (15th) session could be offered with approval by the Clinical Supervisor if the participant was within 9 months of the start of the intervention.

The contingency management component of the intervention allow participants to earn financial incentives. Participants earned incentives for meeting with their peer mentors. They could also earn incentives for engaging in activities related to the study outcomes, such as HIV and STI prevention (e.g., testing, PrEP) or substance use treatment (e.g., 12-step meetings or meetings with a caseworker). Participants could earn incentives multiple times for certain activities, such as 12-step meetings, since these types of activities were expected to be repeated. Participants could also earn incentives for miscellaneous activities related to passport goals or other health-promoting activities. To earn the incentive, participants reported each activity to their peer mentor and completed a short survey providing feedback on their experience with the service/activity. Participants were encouraged, but not required, to provide documentation of completed activities to their peer mentor and did so for approximately half of the activities. Initial incentives were $15 per successful completion of most activities, or $10 for 12-step meetings, with total maximum earnings of $500. In January 2022, incentive amounts were increased 20% to $18 and $12 to address the economic inflation occurring at that time, and the maximum was raised to $600. Participants currently enrolled received retroactive compensation for previously earned incentives as a lump sum. To facilitate comparison across individuals enrolled at different times in the study, in this manuscript the increased incentive amounts are used to present earnings for all individuals.

The Geopass app provided an interface for searching for local providers, as well as the ability to save scheduled appointments, complete the service feedback surveys, review and update goals and steps for reaching them, and track incentive earnings. Many participants found the initial version of the app difficult to use, and a range of technical issues limited its use off and on over periods of the study. Workarounds using REDCap to implement some app functions helped address these issues. The app underwent extensive development during the study in response to user feedback, and its use by participants was much more consistent after its relaunch in January 2023. However, just 36 participants were able to use the revamped version.

### Data Analysis

We present descriptive statistics for baseline characteristics and intervention-related metrics. Total time in study was calculated as the time from the start date to completion of final assessment or last contact with study staff, if the assessment was not completed. Time in study was rounded up to 1 month for individuals whose last contact was within a month of the start date.

Study outcomes were PrEP use since last interview, HIV testing, STI testing, HCV testing, regular place for care, frequent substance use, and recidivism. Because of the distribution of the longitudinal PrEP use outcome in which over half of the participants never used PrEP, a standard logistic random effects regression model was not appropriate. Instead, we modeled PrEP use over time with a group-based trajectory model using the SAS procedure *PROC TRAJ*. The model is a logistic random effects mixture model which identifies distinct subgroups based on the patterns of their outcomes over time. We included arm (MEPS vs. control) as a factor in the model to determine how being in the MEPS group changed the likelihood of being in each PrEP use subgroup. We considered models with two and three groups. The model with two groups had the lowest AIC/BIC and was selected to report here.

We used logistic random intercept models to model the binary outcomes HIV testing, STI testing, frequent substance use, and recidivism, with the random intercept used to account for correlations within participant measurements. Each model included the interaction term between visit and arm (MEPS vs. control) to assess differences between the groups over time and also controlled for location of enrollment (jail vs. community) and time since enrollment. Associations with recidivism were not assessed at baseline.

The outcomes HCV testing and having a regular place for care were only expected to occur once during the study. A participant was defined as achieving the outcome if they endorsed it at a minimum of one follow-up assessment. We used Poisson regression to compare arms, adjusting for location of enrollment, and including an offset for the natural log of follow-up time. Follow-up time was defined as the length of time from baseline until first achievement of the outcome, either date of HCV test or date of follow-up when regular care was endorsed, or until the final completed follow-up in the case of a negative outcome. Individuals reporting an HCV test or having a regular place for care since their release at baseline were excluded from the respective analysis.

SAS 9.4 was used for all analyses and an alpha of 0.05 was used to determine statistical significance.

## Results

**Fig. 2:**
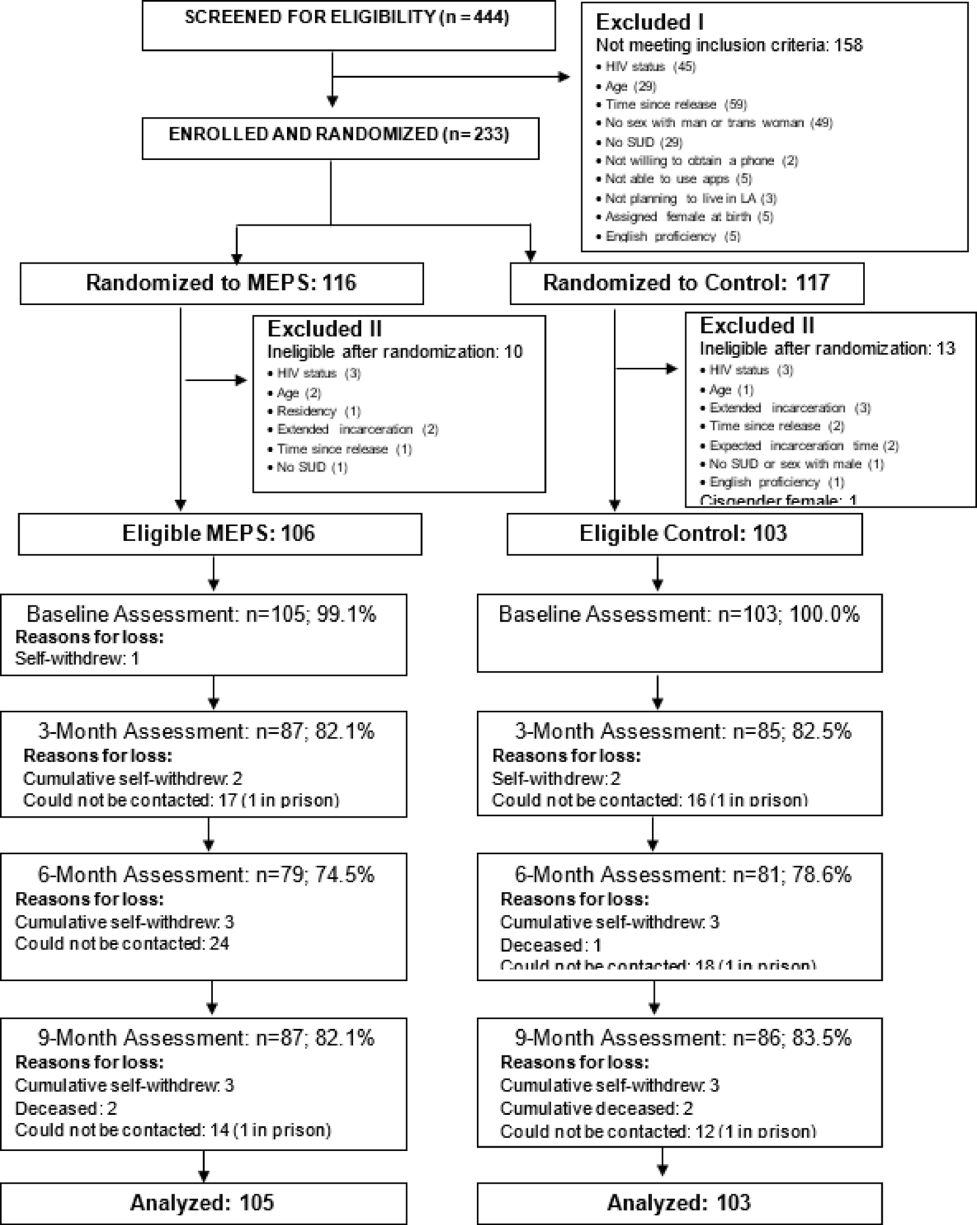
CONSORT chart describing participant screening, enrollment, and follow-up for the Mobile Enhanced Prevention Support Study

Of the 444 potential participants that were screened for eligibility, 286 were found to be eligible (Fig. 2). Of these, 233 were enrolled and randomized into the two arms: 116 to MEPS (intervention arm) and 117 to the control arm. During the study, 23 enrolled participants were determined not to meet the eligibility criteria, resulting in a total of 210 eligible participants. One cis-gender female enrolled due to incomplete inclusion criteria and one individual in the intervention group who withdrew prior to completing all baseline procedures were removed from all analyses, leaving 105 and 103 participants in the MEPS and control arms, respectively. Of the 208 individuals analyzed, 72 (35%) were recruited through referrals (31 MEPS; 41 control). Within the MEPS arm, 82.1%, 74.5% and 82.1% of participants completed the 3-, 6- and 9- month assessments. In the control arm, 82.5%, 78.6% and 83.5% of participants completed the 3-, 6- and 9- month assessments (Fig. 2). The majority of those not interviewed were not able to be contacted, and 6 withdrew and 4 were deceased at the time of follow-up.

Participants predominantly identified as male (85.6%); 9.1% identified as a trans woman and 5.3% as another gender identity (Table 1). 42% of the sample were Hispanic or Latino, 25.5% were Black or African American, 21.2% were White, and 11.5% were of other racial identities. The most common sexual identity was bisexual/pansexual (43.2%), followed by homosexual, gay, or same gender loving (26.7%), and heterosexual (20.9%). Participant ages ranged from 19 to 49, with a mean age of 34 (SD = 7.2). 29% of participants did not complete high school, 41.4% received a high school diploma or GED, and 29.8% had education beyond a high school diploma. 24% had spent time in foster care.

**Table 1:** Participant characteristics (*n* = 208)

	All participants	MEPS	Control
	%	%	%
Gender identity			
Cisgender male	85.6	83.8	87.4
Trans female	9.1	10.5	7.8
Gender non-conforming or non-binary	4.3	4.8	3.9
Other	1.0	1.0	1.0
Age			
18–29	31.3	34.3	28.2
30–39	45.7	47.6	43.7
40–49	23.1	18.1	28.2
Race/ethnicity			
American Indian or Alaska Native	1.0	1.0	1.0
Asian	1.9	1.9	1.9
Black or African American	25.5	25.7	25.2
Hispanic or Latino	41.8	38.1	45.6
Native Hawaiian or Other Pacific Islander	1.4	2.9	0.0
White	21.2	24.8	17.5
Other (usually multiple)	7.2	5.7	8.7
Sexual Identity			
Heterosexual or straight	20.9	17.1	24.8
Homosexual, gay, or same gender loving	26.7	30.5	22.6
Bisexual, pansexual, etc.	43.2	41.0	45.5
Other	9.2	11.4	6.9
Spent time in foster care	23.6	28.6	18.5
Educational Attainment			
Did not complete high school	28.9	29.5	28.2
High school diploma or GED	41.4	38.1	44.7
Tech school or some college	25.5	29.5	21.4
College graduate or higher	4.3	2.9	5.8
Currently a student	8.2	8.6	7.8
Currently employed for wages	11.5	9.5	13.6
Monthly income			
Less than $500	78.2	78.9	77.5
$500-$999	9.7	7.7	11.8
$1,000 or more	12.1	13.5	10.8
Financial situation			
It is not enough to meet basic expenses	86.5	84.8	88.4
It meets needs with little or nothing left	9.1	9.5	8.7
It is enough to live comfortably.	4.3	5.7	2.9
Spent one night or more without a regular place to stay in last 3 months	72.1	74.3	69.9
Lifetime incarcerations			
1	10.6	13.5	7.8
2–10	57.5	59.6	55.3
11–20	21.3	16.4	26.2
21+	10.6	10.6	10.7
Spent time in a juvenile detention facility	25.5	23.8	27.2
Total time incarcerated in lifetime			
< 1 year	26.2	32.7	19.6
1–5 years	41.3	39.4	43.1
6 or more years	32.5	27.9	37.3
Time since release on enrollment			
in custody	9.6	9.5	9.7
< 1 month ago	21.2	21.9	20.4
1-<7 months ago	52.4	55.2	49.5
7–12 months ago	16.8	13.3	20.4

The majority of participants reported monthly earnings under $500 (78.2%), that their earnings were insufficient for basic needs (86.5%), and 72.1% reported being unhoused in the last 30 days. Most (52.4%) participants were enrolled between 1 and 6 months after their release from custody; 9.6% were enrolled while in custody. The vast majority (89.4%) reported prior incarcerations, and 25.5% reported spending time in a juvenile detention facility. Characteristics did not differ greatly between study arms.

Table 2 displays metrics of engagement with the study. Almost all participants (95%) were able to complete passport development. To encourage retention, participants were asked to make contact with a study member on at least a monthly basis or a team member reached out to them. Participants averaged 7.7 (SD = 3.1) monthly check-ins, with those in the MEPS group having more check-ins compared to control [MEPS 8.4 (SD = 3.2), control 6.9 (SD = 2.8), t = 3.5, *p* < 0.001]. MEPS participants averaged far more total contacts with the study than control participants (MEPS: 29.2 SD = 13.1; control: 16.0 SD = 6.5) and were in the study longer (MEPS: 11.7 months SD = 4.8; control: 9.3 SD = 3.8).

**Table 2:** Participation in study and intervention

	All participants	MEPS	Control
Passports completed: # (%)	198 (95%)	101 (96%)	97 (94%)
Months in study: Mean (SD)*	10.5 (4.5)	11.7 (4.8)	9.3 (3.8)
Monthly check-ins completed: Mean (SD)*	7.7 (3.1)	8.4 (3.2)	6.9 (2.8)
Total contacts with study: Mean (SD)*	22.6 (12.3)	29.2 (13.1)	16.0 (6.5)

*Indicates significant difference between MEPS and control

On average, MEPS participants earned $412 (SD=$200; median = $486) in incentives. 35% earned the maximum amount allowed ($600). The most common activities for which MEPS participants earned incentives were peer sessions and substance use treatment activities (e.g. 12-step meetings or SUD case management), due to their being frequently repeated. 92% (*n* = 97) of participants completed at least one session with their peer. For those completing any sessions, the average number completed was 9.2 (SD = 4.2; median = 10). 20% (*n* = 21) completed all 14 sessions, and of those, 4 participants completed a supplemental 15th session. MEPS participants received incentives for a mean of 7.8 (SD = 5.9) substance use treatment activities (e.g. 12-step meetings, or SUD case management) over the course of the study. Excluding peer sessions and substance use treatment, the most common service that participants engaged in was mental health services, comprising 21% of all remaining incentives earned (Table 3), followed by HIV testing and job-related services, which comprised 14% and 9.3%.

**Table 3:** Types of incentives earned, excluding incentivized peer sessions and 12-step meetings (*N* = 917)

Incentive	# (%)
Mental health	193 (21%)
HIV test	124 (14%)
Job related	85 (9.3%)
Government assistance	55 (6.0%)
STI test	50 (5.5%)
Diversion	48 (5.2%)
Vaccines and meds	46 (5.0%)
PrEP evaluation	40 (4.4%)
Begin PrEP	37 (4.0%)
Medical visit	30 (3.3%)
PrEP education	29 (3.2%)
Link to PCP	26 (2.8%)
housing	25 (2.7%)
HCV test	23 (2.5%)
transportation	20 (2.2%)
Other	17 (1.9%)
Exercise	15 (1.6%)
School related	14 (1.5%)
Dental	14 (1.5%)
Sobriety maintenance	11 (1.2%)
Benefits counseling	10 (1.1%)
Gender affirming care	5 (0.5%)

**Fig. 3:**
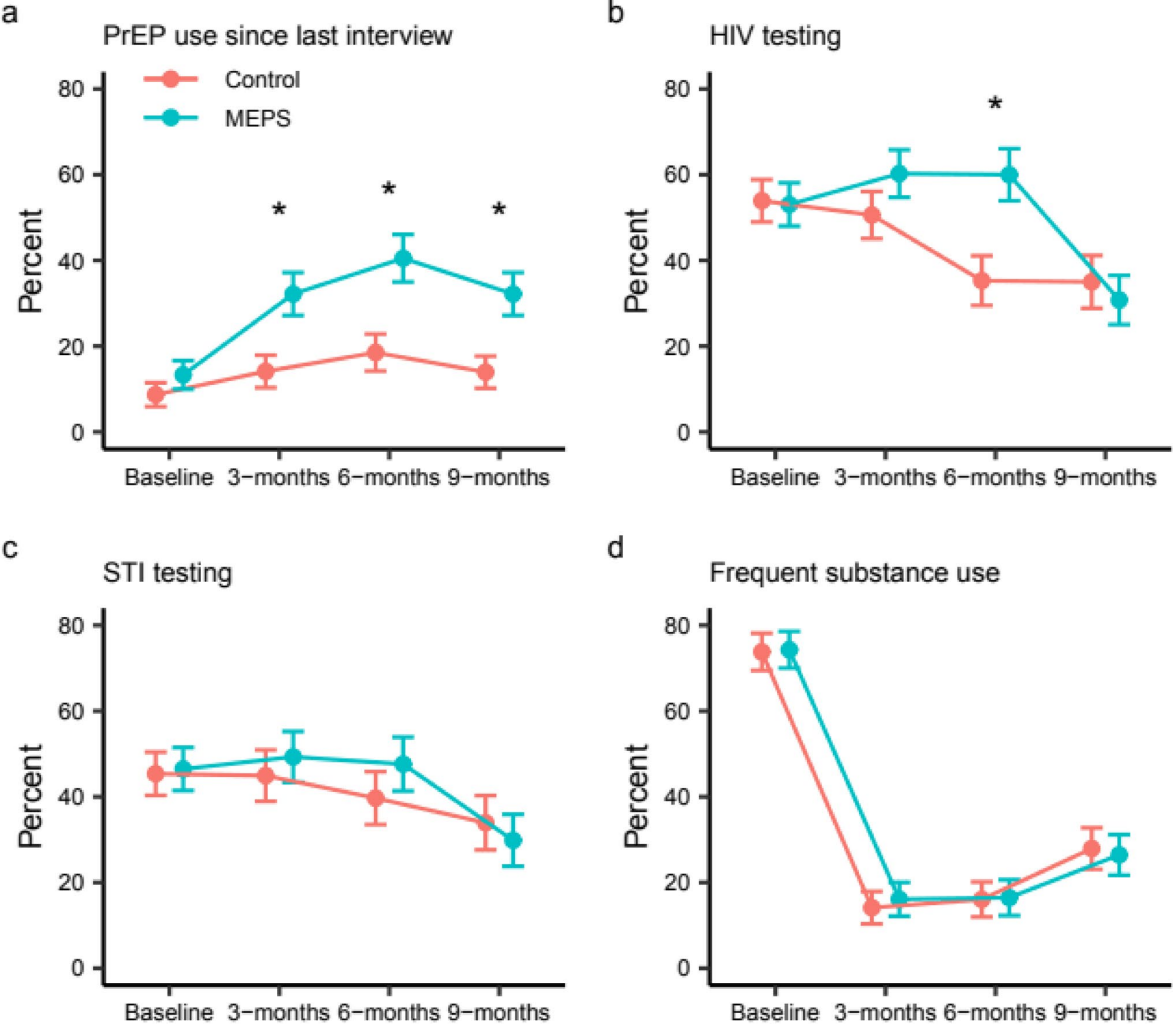
Raw outcomes across time. Percents endorsing each outcome are shown by study arm for (a) PrEP use since last interview, (b) HIV testing, (c) STI testing and (d) frequent substance use at baseline and follow-up assessments. Markers depict percents of individuals and error bars depict standard error. Asterisks indicate significant differences between arms at the given time point based on two-sample tests of equal proportions

**Fig. 4:**
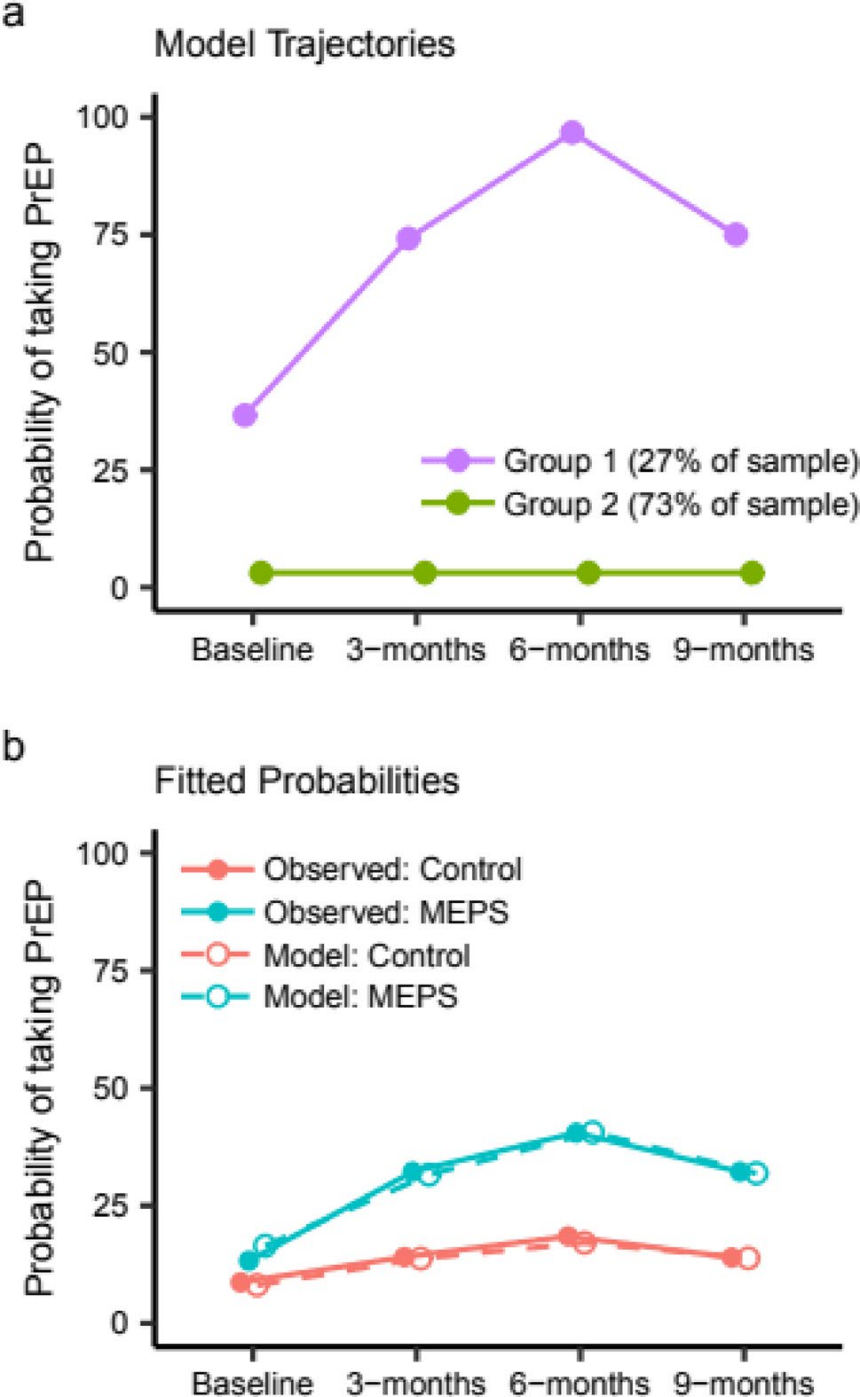
Group-based trajectory model for PrEP use over time. (A) The trajectory model identified two groups of participants: those who used PrEP at some point during the study (Group 1: purple) and those who never used PrEP (Group 2: green). (B) The probability of individuals in the intervention group (blue) and control group (orange) taking PrEP. Probabilities observed in the data are represented by the solid lines and the probabilities determined by the trajectory model are represented by the dashed lines

Figure 3 shows outcomes over time for the control arm and the MEPS arm. A greater percentage of MEPS participants than control participants reported PrEP use since last interview at every time point. At 6 months, 40.5% of MEPS participants had used PrEP compared to just 18.5% of control participants; and at 9 months, 32.2% of MEPS participants had used PrEP, compared to 14.0% of control participants. In the longitudinal trajectory model, we found two groups of PrEP use over time: one group of individuals that never used PrEP, and a second group of individuals who used PrEP at some point during follow-up (Fig. 4a). The odds of a MEPS participant being among those who used PrEP was 3.8 times the odds of a control participant (95% CI = 1.8, 8.0). The fitted probability of participants who used PrEP based on the trajectory model was similar to the observed frequencies of PrEP use for both MEPS and control participants (Fig. 4b).

Being in the group that used PrEP was associated with identifying as homosexual, gay, or same gender loving (40%) or another sexual identity (36.8%) (*p* = 0.016) and identifying as a trans woman (63.2%) (*p* < 0.001), but negatively associated with identifying as Black or African American (20.8%; *p* = 0.028) and length of lifetime incarceration (*p* = 0.036). PrEP users reported significantly more male partners (mean = 3.5, median = 1, IQR = 4 vs. mean = 1.0, median = 0, IQR = 1) and a greater frequency of anal sex (mean = 2.4 times, median = 0, IQR = 2 vs. mean = 1.6, median = 0, IQR = 0) in the last 3 months at baseline compared to those who never used PrEP (Wilcoxon *p* < 0.001).

There were higher levels of recent HIV testing in the MEPS arm at 6 months compared to the control arm (MEPS arm: 59.4%, control arm: 35.3%, *p* = 0.004), but similar levels of recent testing at 9 months (MEPS arm: 31.8%, control arm: 35.6%, *p* = 0.662). From the random effects logistic regression model, the change from baseline to 6 months (Table 4; AOR = 3.5, 95% CI = 1.3, 9.5, *p* = 0.014) was significantly different between the arms. The percent of participants reporting recent STI testing was similar over time between MEPS and control (Fig. 3; Table 4). The proportion of individuals reporting recent HCV testing increased in the control arm from 32% at baseline to 75% at the end of follow-up, and from 30 to 80% in the MEPS arm, but there was no difference in testing uptake between arms (RR = 1.11; 95% CI = 0.73, 1.72; *p* = 0.620).

**Table 4:** Mixed effects logistic models controlling for time since enrollment and enrollment location

Outcome	AOR	95% CI	pvalue
HIV testing			
3-month interaction	1.64	0.65, 4.12	0.293
6-month interaction	**3.50**	**1.29**,** 9.52**	**0.014**
9-month interaction	1.10	0.38, 3.17	0.867
STI testing			
3-month interaction	1.20	0.45, 3.21	0.721
6-month interaction	1.47	0.52, 4.10	0.465
9-month interaction	0.88	0.28, 2.75	0.827
Frequent substance use			
3-month interaction	1.00	0.32, 3.17	0.995
6-month interaction	0.84	0.27, 2.66	0.769
9-month interaction	0.48	0.17, 1.38	0.174
Recidivism			
6-month interaction	0.44	0.05, 4.23	0.477
9-month interaction	1.17	0.12, 11.77	0.893

**Bold** indicates significance at p < 0.05

Models for PrEP use, HCV testing, and establishing a regular place for care are reported in the text

Frequent substance use, defined as at-least weekly use of illegal or self-identified problematic substances, decreased substantially from 74% at baseline to approximately 15% at 3 months and 6 months, then increased slightly at 9 months, with similar levels of reported use in the MEPS and the control arms (Fig. 3). In a follow-up analysis, we assessed the association between frequent substance use and incentives earned for engagement in SUD treatment services among intervention participants using a logistic random intercept model. Number of SUD treatment incentives earned was negatively associated with frequent substance use at 3 months (AOR = 0.869, 95% CI = 0.780, 0.969, *p* = 0.0473), but not at 6 (AOR = 0.912, 95% CI = 0.815, 1.020, *p* = 0.2128) or 9 months (AOR = 0.941, 95% CI = 0.860, 1.030, *p* = 0.3678).

During the study, 29 (15%) individuals were re-incarcerated. Recidivism was similar between groups, with 16.2% (*n* = 16) of individuals in the MEPS arm and 14.0% (*n* = 13) of individuals in the control arm being re-incarcerated. Large proportions of MEPS and control participants established a regular place for care during the study (MEPS: 90.9%, control: 88.2%), with no statistically significant difference between the groups (RR = 1.07; 95% CI = 0.66, 1.74; *p* = 0.780).

To assess the potential impact of the COVID-19 pandemic on outcomes, we compared outcomes for individuals enrolled prior (58%) to February 25, 2022 with those enrolled after (42%). February 25, 2022 was the date of an executive order ending the majority of California’s stay-at-home orders [52]. Those enrolled during COVID-19 actually had increased odds of having been recently tested for HIV (AOR = 2.63, 95% CI = 1.67, 4.14, *p* < 0.001) and STIs (AOR = 3.00, 95% CI = 1.79, 5.04, *p* < 0.001) and increased RR for HCV testing (RR = 3.16, 95% CI = 1.93,5.17, *p* < 0.001). The interaction terms in these models for group and time point decreased by 0-0.05, but no terms changed significance. Enrollment during COVID-19 was not significantly associated with any other outcomes.

## Discussion

The MEPS intervention robustly increased PrEP use and HIV testing over time in this racially and ethnically diverse sample of individuals assigned male at birth leaving incarceration in Los Angeles. A recent modeling study using empirical data from Black MSM in Chicago indicated a potential population-level impact of interventions that increase PrEP uptake during reentry on HIV incidence among their sexual partners [53], suggesting that interventions using the MEPS approach may also benefit the sex and needle-using partners of those enrolled. The MEPS intervention did not, however, yield positive findings for other tested outcomes (i.e., HCV and STI testing and frequent substance use). Below, we discuss some of the possible reasons for these apparently contradictory findings.

HIV testing and PrEP were likely the most readily available preventive health services for study participants to access. There are numerous free storefront, mobile and clinic-based HIV testing sites in Los Angeles County, as well as self-test kit programs [54] that, unlike most STI/HCV testing services, do not require advance appointments or potentially long wait times [55]. Furthermore, HIV testing and PrEP services are often incentivized, creating a synergy between the MEPS intervention and local service delivery. For example, the study’s main community partner, L.A. CADA, provided HIV testing and PrEP navigation throughout most of the study period, and clients could often receive gift cards for obtaining these services. While both MEPS and control participants in our study were made aware of L.A. CADA’s services, the MEPS group could locate them through the search utility in the intervention’s mobile app, had more opportunities to be reminded of them and learn of the additional incentives from their Peer Mentors. The ready availability of these services likely meant that smaller shifts in attitudes and perceptions were needed to encourage use of them than use of other services. The characteristics of those who used PrEP during the study were similar to those previously shown to be associated with PrEP use at baseline [56], suggesting that additional efforts are necessary to shift attitudes for some groups.

Anecdotally, the Peer Mentors suggested that many MEPS participants did not feel a need for STI testing because they were in residential recovery programs and not sexually active. Despite this, however, some participants indicated a willingness to take PrEP because they came to see it as part of a recovery journey that included taking proactive care of their physical health. However, this motivation did not apply to getting tested for acute STIs like gonorrhea, chlamydia, and syphilis. Finally, HIV but not STI screening is required for individuals on PrEP [57]; and STI screening is not routinely conducted for individuals who initiate PrEP through a pharmacy in California, a practice which became legal in the state in January 1, 2020 [58].

Despite not observing an intervention impact on frequent substance use, substance use declined substantially from baseline in both groups. This is not surprising given that substance use at each follow-up assessment was compared to use in the time prior to participants’ most recent incarceration or, in most cases, their entrance into a residential recovery facility. Given the large magnitude of these declines in reported use in the control group, the MEPS intervention may not have been powerful enough to provide additional benefit on top of the usual care participants received. Furthermore, MEPS participants were incentivized for utilizing substance use-related services, not for reducing substance use. Hence, the MEPS incentives did not align as closely to the substance use outcome as they did for testing or PrEP use. Finally, reducing or abstaining from substance use involves ongoing motivation and behavior change, rather than the periodic behaviors associated with screening or obtaining a prescription and refills [59]. This difference may make sustained reductions in use a more challenging target for intervention.

Efforts to reach those who have not engaged with the HIV prevention continuum at this point in the HIV pandemic require a focus on populations facing multiple social and economic barriers and as well as substance use and mental health challenges that exacerbate one another [60, 61]. We designed our multi-pronged intervention to offer such individuals multiple levels of support for proactive behavior change. Financial incentives can be much less expensive to provide than other types of interventions and have been shown to maintain service program engagement over time [59, 62, 63]. Goal setting, client-tailoring, and motivational interviewing were critical to providing participants with a sense of autonomy, while offering skills that are transferable to the other goals to which they might aspire. Participants’ histories of frequent incarcerations, in addition to their current group-living settings, with restricted freedoms, further the importance of giving participants agency over the intervention process.

### Limitations

This study did have limitations. First, due to the COVID-19 pandemic, several elements of the study design were altered during implementation. Delays resulting from the closures and uncertain and changing policies resulted in a smaller sample than initially planned (*n* = 208 vs. 266). In addition, these closures and new policies likely limited some participants’ access to services including HIV/STI/HCV testing and PrEP. The virtual Mentor-participant interactions that replaced in-person meetings for much of the pandemic may have limited the ability to establish rapport with some participants, which could have decreased the efficacy of the intervention or contributed to attrition. Additionally, challenges with the mobile app early in the study make it difficult to assess its contribution to the efficacy of the intervention. Because MEPS was a multi-pronged intervention that included peer mentors, contingency management, and a mobile app, Geopass, it was impossible to disambiguate which element of MEPS were most effective. Because the elements of the intervention support one another, we suspect the combination of the elements synergistically support positive behavior change; in particular, the app was designed to facilitate the other aspects of the intervention. However, we did not test this hypothesis directly.

### Future Research

The one-on-one and individually tailored nature of the MEPS intervention makes adaptation for many other populations highly feasible. For example, our team, with input from community partners and a community advisory board, is in the process of adapting MEPS for people assigned male at birth in three new groups: people who inject drugs, who speak Spanish, and who live in less urban settings. Adaptations for other genders are another promising avenue but should address the reproductive health concerns, sexual trauma, and child welfare issues affecting many cisgender women and individuals of transmasculine experience with involvement in the criminal legal system [64–67]. Biomedical advances also present additional opportunities for future implementation. For example, use of DoxyPrEP, a short treatment with antibiotics to prevent STIs, should be considered in future interventions of this sort. Expansion of interventions such as MEPS, along with efforts to increase MEPS’ impact on substance use and HCV/STI testing, is critical to the goal of ending the HIV epidemic.

## Electronic Supplementary Material

Below is the link to the electronic supplementary material.

Supplementary Material 1
