# Supplementary material for: Randomized Controlled Trial Testing an HIV/STI Prevention Intervention Among People Leaving Incarceration Who Were Assigned Male at Birth, Have Sex with Men and A Substance Use Disorder
Source: AIDS Behav. 2025 Jul 10;29(12):3806–21. doi: 10.1007/s10461-025-04818-4 (PMC12580433; doi:10.1007/s10461-025-04818-4)
Supplement: Supplementary file 1 — Supplementary Material 1 [file 10461_2025_4818_MOESM1_ESM.docx]

Supplemental methods

Non-outcome measures

For gender, race/ethnicity, and sexual identity, participants could select as many categories as they felt applied. Options for gender included female, male, transgender female, transgender male, non-binary or gender non-conforming, and other. Options for race/ethnicity included American Indian, Black/African American, White, Latino/Hispanic, Pacific Islander, and other. Those who selected multiple options were asked a follow-up question, “Which of your backgrounds do you identify with most strongly?” The selected response was used as the primary background. Those responding “I identify equally with all of my backgrounds” were considered multi-racial. Options for sexual identity were straight/heterosexual, bisexual, gay or homosexual, same gender loving, queer, and other. Several wrote in “pansexual” or “open” as their selected sexual identity, and these individuals were grouped with individuals who identified as bisexual into a single bisexual/pansexual category. Options for educational attainment were “Eighth grade or less than eighth grade”, “More than eighth grade but did not complete High School”, “GED”, “High School Diploma”, “Tech School or Some College”, and “College graduate or higher”. To reduce small cell counts, we collapsed the categories that indicated less than high school diploma into a single category, “Did not complete high school”, and collapsed the categories “High School Diploma” and “GED” into a single category.

We assessed housing instability by asking “In the last 3 months, did you ever spend one night or more without a regular place to stay?” with response options yes or no. To assess financial situation, we asked for an estimate of monthly income with response options “Less than $500”, “$500-$999”, “$1,000 - $1,999”, “$2,000 - $2,999”, “$3,000 - $3,999”, and “$4,000 or more”. For analysis, we collapsed responses in the upper four categories to a single category, “$1,000 or more”. We also asked participants, “How would you describe your overall personal financial situation when in the community” with response options “It is enough to live comfortably”, “It meets needs with a little left”, “It just meets basic expenses”, and “It is not enough to meet basic expenses”. We present the variable Financial Situation with the categories “It meets needs with a little left” and “It just meets basic expenses” collapsed into a single category, “It meets needs with little or nothing left”.

We asked several questions regarding incarceration history. Participants were asked for a numeric estimate of total number of lifetime incarcerations, which we categorized into 1, 2-10, 11-20, and 21+. We also asked for an estimate of total time spent incarcerated, with response options “Less than 1 week”, “7-30 days”, “1-5 months”, “6-11 months”, “1-2 years”, “3-5 years”, and “6 or more years”. We collapsed responses into the following categories, “<1 year”, “1-5 years”, and “6 or more years”. Participants were asked about the types of facilities where they had spent time. Here, we report specifically on endorsement of having spent time in a juvenile detention facility. Finally, we asked for the release date from their most recent incarceration, calculated the time since release at enrollment, and categorized to in custody, “<1 month ago”, “1-<7 months ago”, and “7-12 months ago”.
